# Comparing Changes in FEV_1_ and Impulse Oscillometry Parameters Following Methacholine Challenge Testing: Physiological Correlates, Clinical Markers, and Pulmonary Symptoms

**DOI:** 10.3390/jcm15052025

**Published:** 2026-03-06

**Authors:** Thomas Ringbaek, Lars Frølund, Jann Mortensen, Charlotte S. Ulrik, Laura H. Thomsen, Henrik H. El Ali

**Affiliations:** 1 Allergy and Lung Clinic Helsingør, Sct. Olai Gade, 3000 Helsingør, Denmark; thomasringbaek@gmail.com (T.R.); lars.froelund@dadlnet.dk (L.F.); 2 Department of Clinical Physiology and Nuclear Medicine, Copenhagen University Hospital-Rigshospitalet, 2100 Copenhagen, Denmark; jann.mortensen@regionh.dk; 3 Department of Medicine, The National Hospital, 100 Torshavn, Faroe Islands; 4 Faculty of Health Sciences, University of the Faroe Islands, 100 Torshavn, Faroe Islands; 5 Institute of Clinical Medicine, Faculty of Health and Medical Sciences, University of Copenhagen, 1165 København, Denmark; csulrik@dadlnet.dk; 6 Respiratory Research Unit Hvidovre, Department of Respiratory Medicine, Copenhagen University Hospital—Hvidovre, 2650 Hvidovre, Denmark; laurahohwu@gmail.com; 7 Department of Biomedical Sciences, University of Copenhagen, 1165 København, Denmark

**Keywords:** impulse oscillometry, methacholine challenge, airway hyperresponsiveness, FEV_1_, R_5_, small airways, symptoms, FeNO, asthma control

## Abstract

**Background:** Spirometry-based methacholine challenge testing using the provocative dose causing a 20% decline in forced expiratory volume in 1 s (FEV_1_, PD_20_) is a reference method for assessing airway hyperresponsiveness. Impulse oscillometry (IOS), performed during tidal breathing, may capture airway mechanical changes not fully reflected by spirometry. We compared FEV_1_- and IOS-based methacholine responsiveness in a large, real-world adult cohort and examined associations with clinical markers and symptoms. **Methods:** We analyzed 794 consecutively referred adults undergoing standardized methacholine challenge testing with concurrent spirometry and IOS. IOS positivity was defined as a ≥40% increase in resistance at 5 Hz (ΔR_5_ ≥ 40%). Agreement between FEV_1_–PD_20_ positivity (PD_20_ ≤ 1440 µg) and IOS positivity was evaluated using cross-classification and Cohen’s κ. Associations between continuous responses were assessed using Pearson and Spearman correlations. The relationship between ΔR_5_ and the probability of a ≥20% decline in FEV_1_ was examined using logistic regression. Predictors of ΔR_5_ were assessed using multivariable linear regression. Symptom severity was recorded immediately post-challenge using a five-point Likert scale and related to physiological responses. **Results:** FEV_1_–PD_20_ classified 37.5% of participants as hyperresponsive, whereas IOS positivity (ΔR_5_ ≥ 40%) classified 70.6%. Agreement between methods was limited (κ = 0.09; *p* < 0.01). ΔFEV_1_ and ΔR_5_ were weakly correlated (r = −0.287; ρ = −0.306; both *p* < 0.001; R^2^ = 0.08). A 20% decline in FEV_1_ corresponded on average to a 74% increase in R_5_, whereas ΔR_5_ ≥ 40% corresponded to an average FEV_1_ decline of 7.6%. In multivariable models, referral diagnosis group and age independently predicted ΔR_5_, whereas FeNO and baseline FEV_1_% predicted did not. Baseline FEV_1_% predicted modified the ΔFEV_1_–ΔR_5_ slope (interaction β = −0.0317; *p* = 0.0028). Post-challenge symptom (5-point Likert) related to MCT was associated with both ΔFEV_1_ and IOS responses; ΔFEV_1_ showed a stronger linear association with symptoms, whereas IOS measures showed larger stepwise differences across symptom categories. **Conclusions:** IOS identifies a larger, partly distinct subset of methacholine-responsive individuals compared with conventional FEV_1_–PD_20_ criteria and detects mechanical changes at lower levels of spirometric impairment. Despite limited concordance, IOS provides complementary physiological and symptom-relevant information when used alongside spirometry. Standardized IOS response definitions and prospective validation are needed to establish clinical utility.

## 1. Introduction

Airway hyperresponsiveness is a hallmark feature of asthma and may also [1] occur in other lung disorders such as chronic obstructive pulmonary disease (COPD), bronchiectasis, and post-infectious airway disease [2,3]. The methacholine challenge test (MCT) is a well-established method to assess airway hyperresponsiveness, most commonly by identifying the provocative dose (or concentration) that induces a ≥20% fall in forced expiratory volume in 1 s (FEV_1_), denoted PD_20_ (or PC_20_).

Over the past decade, impulse oscillometry (IOS) has emerged as a surrogate or complementary technique to spirometry for conducting MCT in both adults and children [4,5]. IOS measures respiratory system impedance during tidal breathing and therefore eliminates the need for forced expiratory maneuvers required in spirometry. It provides indices of resistance (R) at different frequencies (e.g., R_5_, R_20_) and reactance (X_5_, AX); the difference R_5_–R_20_ is often interpreted as reflecting peripheral (small-airway) resistance, while more negative X_5_ or larger AX may indicate distal airway dysfunction or reduced compliance.

Several studies suggest that IOS detects methacholine responsiveness earlier or in individuals who do not decline at least 20% in FEV_1_, which might shorten the test and reduce the cumulative dose of methacholine administered [6,7,8,9,10,11,12,13].

Despite this promise, consensus is lacking on optimal IOS thresholds (e.g., percentage increase in R_5_ or specific X_5_/AX shifts) that correspond to conventional PD_20_ definitions, particularly in adult populations. Earlier experimental work in primary children suggested that an ~40% increase in R_5_ might approximate PD_20_-FEV_1_, but correspondence is imperfect and may not generalize across populations [14].

A few studies, primarily involving asthma patients, have evaluated the correlation between changes in IOS parameters and changes in FEV_1_ after MCT, yielding varying results [8,9,11,12,15,16,17,18]. This correlation may depend on specific characteristics, including diagnosis, phenotypes, and markers of disease activity, such as asthma symptoms score, fractional exhaled nitric oxide (FeNO), and baseline FEV_1_.

Recent reviews highlight that IOS can detect abnormalities not captured by spirometry and may improve the assessment of small-airway impairment in asthma and other airway diseases [19]. Small airway impairment is a critical and interesting area of respiratory medicine because it plays a central, early, and often undetected role in the development and progression of COPD and asthma [20]. Adding oscillometry to spirometry improves the identification of uncontrolled asthma [21]. Methacholine-induced increases in R_5_ have been observed in healthy adults before a pronounced fall in FEV_1_ [14], and in adult asthma, combining IOS parameters such as AX with spirometry improves disease discrimination even when spirometry is normal [22]. Moreover, post-methacholine indices reflecting small-airway involvement, including R_5_–R_20_, have been linked to disease severity in selected populations, highlighting the potential clinical relevance of oscillometric responses during bronchial provocation [23].

However, it remains unclear how differences between IOS and spirometry during methacholine challenge translate into patient-reported symptoms. While FEV_1_ represents a global expiratory flow limitation, IOS captures frequency-dependent changes in airway resistance and reactance that may evolve non-linearly and may be perceived differently by patients. Whether IOS responses align more closely with symptom severity than FEV_1_ in adult populations undergoing MCT has not been systematically examined.

## 2. Aims and Study Hypothesis

The primary aim of this study was to compare impulse oscillometry (IOS)–based responsiveness metrics with conventional FEV_1_-based responsiveness in a large, real-world adult cohort undergoing MCT.

We hypothesized that an increase in resistance at 5 Hz of ≥40% (ΔR_5_ ≥ 40%) would show limited correspondence with a decline of ≥20% in FEV_1_, reflecting differences in how IOS and spirometry capture airway responses to methacholine.

Secondary aims were to examine (i) the strength and consistency of the relationship between changes in FEV_1_ and IOS parameters, (ii) clinical and physiological factors associated with the magnitude of the IOS response, and (iii) whether this relationship varied across diagnostic and inflammatory subgroups.

Finally, we hypothesized that IOS parameters would show larger stepwise differences across symptom severity categories during MCT, whereas ΔFEV_1_ would demonstrate a more linear association with symptom severity.

## 3. Methods

### 3.1. Study Design and Population

This was a retrospective, cross-sectional observational study involving adult patients referred to our secondary care Allergy and Lung Clinic between 1 January 2024, and 31 December 2024, for evaluation of asthma or suspected asthma.

**Figure 1:**
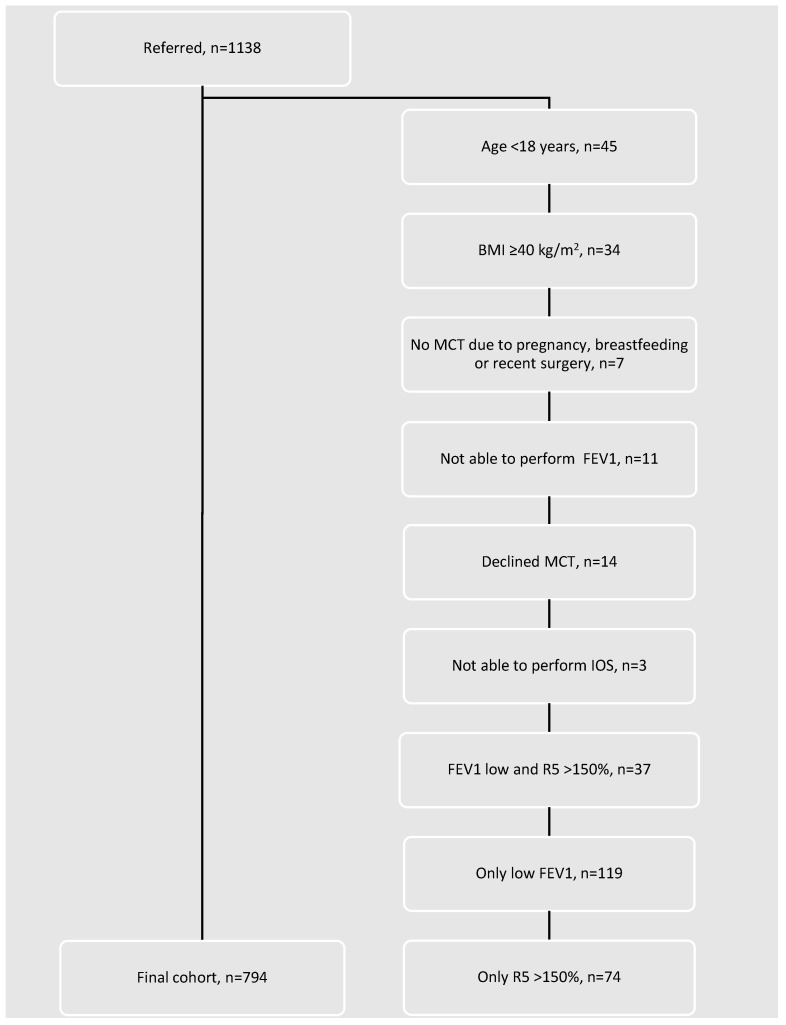
Flow diagram of patient recruitment and exclusions. Of 1138 adults referred for methacholine challenge testing, exclusions were applied sequentially due to age < 18 years, body mass index ≥ 40 kg/m^2^, contraindications to methacholine challenge, inability to perform spirometry or impulse oscillometry, or patient refusal. Additional exclusions were applied for abnormal baseline lung function (FEV_1_ below the safety threshold for methacholine challenge and/or baseline R5 > 150% predicted). The final study cohort comprised 794 participants.

A total of 1139 patients underwent screening, and 794 patients fulfilled the inclusion criteria and were included in the final analysis (Figure 1).

### 3.2. Inclusion Criteria

1. Clinical symptoms suggestive of lung disease (intermittent or persistent cough, dyspnea, wheeze, or chest tightness).
2. Eligibility for MCT defined as:
  a. FEV_1_ ≥ 1.5 L and ≥ 60% of predicted;
  b. Non-pregnant and not breastfeeding;
  c. Baseline R_5_ < 150% of predicted.
3. Age ≥ 18 years.
4. Ability to perform both spirometry and IOS.

### 3.3. Exclusion Criteria

1. Body mass index (BMI) ≥ 40 kg/m^2^.
2. Recent myocardial infarction, unstable angina, uncontrolled hypertension, or other conditions that could be aggravated by increased intrathoracic pressure (e.g., pneumothorax or thoracic surgery within 4 weeks).
3. Hemoptysis of unknown origin, active tuberculosis, or recent eye or brain surgery (within 4 weeks).
4. Recent bronchodilator or leukotriene receptor antagonist use: short-acting β_2_-agonists within 6 h, long-acting β_2_-agonists within 48 h, short-acting antimuscarinics within 12 h, long-acting antimuscarinics within 7 days, or montelukast within 3 days prior to MCT.

### 3.4. Lung Function and Physiological Measurements

Spirometry was performed using Medical Electronic Construction (M.E.C.) equipment (M.E.C. Respiratory Diagnostics, Brussels, Belgium) in accordance with ATS/ERS guidelines [24]. FeNO was measured using the NObreath™ device (Bedfont Scientific Ltd., Maidstone, UK). In all cases, FeNO and IOS measurements were obtained before spirometry.

### 3.5. Impulse Oscillometry

Respiratory system impedance was measured using the Vyntus™ IOS (Vyaire Medical, Höchberg, Germany) with SentrySuite v3.20 software, following the standard protocol from the ERS Task Force on Respiratory Impedance [25]. The system applies pressure oscillations of multiple frequencies during quiet breathing to estimate respiratory resistance (Rrs) and reactance (Xrs) [26]. Reference values for Rrs and Xrs were derived from established prediction equations accounting for posture and ethnicity [27].

The primary IOS endpoint was ΔR_5_%, defined as the percent change in total respiratory system resistance at 5 Hz from baseline to post-methacholine. As secondary oscillometric measures, we evaluated AX (reactance area) and R_5_–R_20_ as indices commonly linked to peripheral airway involvement and frequency dependence of resistance. IOS was performed before spirometry and repeated at least 10 min after the final FEV_1_ measurement during the MCT.

### 3.6. Methacholine Challenge Test (MCT)

Bronchial hyperresponsiveness was evaluated using a five-step dosimeter protocol with the Aerosol Provocation System (APS) (Vyaire Medical GmbH, Höchberg, Germany) employing a single methacholine concentration of 25 mg/mL. The APS automatically calculated the cumulative delivered dose (PD_20_) in SentrySuite v3.20 software [28].

MCT was stopped when FEV_1_ had decreased 20% (considered a positive test) or maximum dose of 1440 µg was delivered. Short- and long-acting bronchodilators were withheld as per exclusion criteria.

### 3.7. Clinical and Questionnaire Data

Respiratory symptoms were assessed using the 6-item Asthma Control Questionnaire (ACQ-6), excluding the FEV_1_% predicted item. Each item is scored from 0 to 6, with higher values indicating poorer control. For analyses, the average score (range 0–6) was used and summarized as median [IQR] due to non-normal distribution [29]. In addition, participants were categorized into three ACQ-6 control strata to facilitate categorical comparisons:

1: Well-controlled asthma: a score <0.75
2: Partly controlled asthma: scores between 0.75 and 1.5
3: Uncontrolled asthma: a score >1.5

### 3.8. Post-Test Symptom Assessment and Grouping

Immediately after completion of the methacholine challenge, patients were asked to rate how their breathing felt compared with before the MCT using a five-point Likert symptom scale:

1 = “I feel much better”,
2 = “I feel a little better”,
3 = “I feel no change”,
4 = “I feel a little worse”,
5 = “I feel much worse”.

This response was recorded in the categorical variable symptom group with the corresponding labels (“much better”, “a little better”, “no change”, “a little worse”, “much worse”). Symptom data was available for 791 of 794 participants (3 missing responses, excluded listwise from the symptom-based analyses).

### 3.9. Diagnostic Classification

Patients were classified based on clinical evaluation and follow-up within 6 months after the index visit into five groups:

1. Asthma only—diagnosis of asthma without COPD or other major lung disease.
2. Asthma + COPD—concurrent diagnoses of asthma and COPD.
3. COPD only—diagnosis of COPD without asthma or other major lung disease.
4. Other lung diseases—including interstitial lung disease, bronchiectasis, post-COVID syndrome, or infection.
5. Non-lung conditions—e.g., rhinitis, sinusitis, dysfunctional breathing, or gastroesophageal reflux.

### 3.10. Statistical Analysis

All analyses were performed using R (version 4.5.1; R Foundation for Statistical Computing, Vienna, Austria). Continuous variables are presented as mean ± SD or median (min–max), as appropriate, and categorical variables as counts and percentages. Group comparisons were conducted using Student’s *t*-test or Wilcoxon rank-sum test for continuous variables and χ^2^ test or Fisher’s exact test for categorical variables, depending on data distribution and cell counts.

Associations between continuous methacholine responses were assessed using Pearson’s correlation coefficient (r) for approximately normally distributed variables and Spearman’s rank correlation coefficient (ρ) otherwise. Agreement between binary classifications based on spirometry and oscillometry—defined as FEV_1_–PD_20_ positivity (≤1440 µg) and IOS positivity (ΔR_5_ ≥ 40%), respectively—was evaluated using Cohen’s κ with 95% confidence intervals.

To examine the relationship between ΔR_5_ and the probability of a ≥20% fall in FEV_1_, logistic regression was used, with predicted probabilities and 95% confidence intervals presented graphically. ΔFEV_1_ (%) was defined as 100 × (post − baseline)/baseline (negative values indicate a decline).

Factors associated with the magnitude of the oscillometric response were examined using multivariable linear regression, with ΔR_5_ (%) as the dependent variable and adjustment for baseline FEV_1_ (% predicted), age, sex, body mass index (BMI), FeNO, and diagnostic group. Model assumptions were evaluated by inspection of residuals.

To assess whether the relationship between ΔFEV_1_ and ΔR_5_ differed across clinical subgroups, interaction terms were tested within multivariable linear models. Stratified correlation analyses were performed for descriptive purposes only.

Associations between post-challenge symptom severity (ordinal scale) and physiological responses were evaluated using Kruskal–Wallis tests, with Dunn post hoc tests applied when overall differences were detected. To formally compare the strength of correlations between symptom severity and different physiological measures sharing a common variable, Steiger’s test for dependent correlations was used.

All statistical tests were two-sided, and a *p*-value <0.05 was considered statistically significant. Effect sizes were reported alongside statistical significance where relevant.

## 4. Results

### 4.1. Study Population

A total of 794 adults referred for MCT were included. Mean age was 53 ± 18 years, and 63% were women. Baseline lung function was largely preserved, with a mean FEV_1_ of 92.9 ± 13.4% predicted, while pre-challenge R_5_ averaged 91.1 ± 23.3% predicted. Baseline demographic and physiological characteristics stratified by diagnostic group are presented in Table 1.

### 4.2. Relationship Between Changes in FEV_1_ and R_5_ During Methacholine Challenge

**Figure 2:**
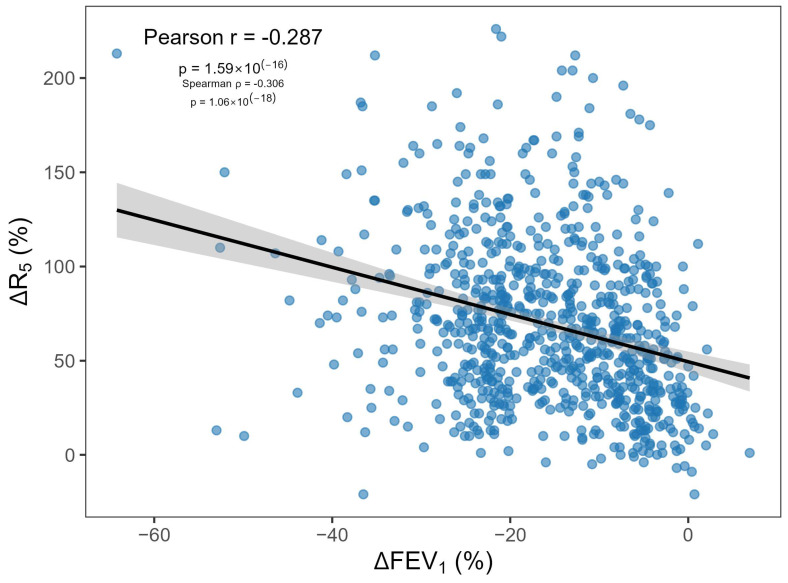
Relationship between methacholine-induced changes in spirometry and oscillometry. Scatter plot of percentage change in FEV_1_ (ΔFEV_1_, %; negative values indicate a fall) versus percentage change in R5 (ΔR5, %) measured during methacholine challenge testing. The solid line shows the fitted linear regression with 95% confidence band.

Across the cohort, the percentage fall in FEV_1_ (ΔFEV_1_; negative values indicate a decline; positive values indicate an increase) was weakly and inversely associated with the percentage rise in R_5_ (ΔR_5_) during methacholine challenge (Pearson r = −0.287; Spearman ρ = −0.306; both *p* < 0.001; Figure 2). Despite statistical significance, the explained variance was low (R^2^ = 0.08), indicating substantial inter-individual variability.

Linear regression showed a modest slope (−1.24), such that a 20% fall in FEV_1_ corresponded on average to a 74% rise in R_5_, whereas the commonly used IOS threshold of ΔR_5_ ≥ 40% corresponded to only a 7.6% fall in FEV_1_.

### 4.3. Comparison of FEV_1_-Based and IOS-Based Responsiveness Criteria

Using 298. participants (37.5%) met the definition of bronchial hyperresponsiveness based on a ≥20% fall in FEV_1_ (PD_20_ ≤ 1440 µg), while 155 (19.5%) met the more stringent PD_20_ ≤ 400 µg threshold. In contrast, 561 participants (70.6%) exhibited an increase in R_5_ ≥ 40%.

Across all diagnostic groups, the proportion of participants classified as positive by the IOS criterion was significantly higher than by the FEV_1_ criterion (Table 2). This pattern was consistent in asthma, COPD, other lung diseases, and in participants without a final lung disease diagnosis.

Cross-classification demonstrated limited concordance between methods, with an overall agreement of 49.6% and Cohen’s κ = 0.09 (*p* < 0.01) (Table 3). Among participants who did not meet the FEV_1_–PD20 criterion, approximately two-thirds were classified as IOS-positive. These findings indicate that IOS and spirometry identify overlapping but distinct patterns of methacholine responsiveness, and that an IOS-positive response frequently occurs in the absence of a ≥20% decline in FEV_1_.

### 4.4. Clinical and Physiological Factors Associated with ΔR_5_

**Figure 3:**
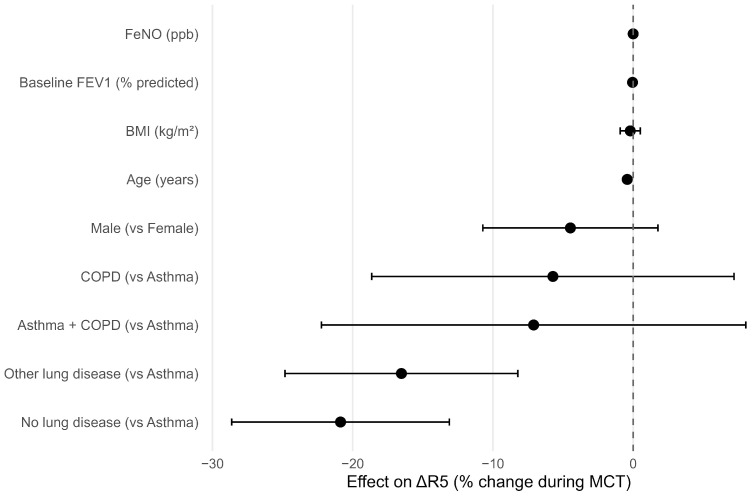
Multivariable predictors of the ΔR5 response to methacholine. Forest plot showing adjusted linear regression coefficients (points) with 95% confidence intervals (horizontal lines) for associations with percent change in R5 (ΔR5) during methacholine challenge testing. Coefficients are adjusted for baseline FEV_1_ (% predicted), fractional exhaled nitric oxide (FeNO), age, sex, body mass index (BMI), and diagnostic group; asthma is the reference category for diagnosis. The dashed vertical line indicates no effect (β = 0); negative coefficients indicate smaller ΔR5 responses relative to the reference.

In multivariable linear regression adjusting for baseline FEV_1_ (% predicted), age, sex, BMI, FeNO, and diagnostic group (Figure 3), diagnosis remained an independent predictor of ΔR_5_ (overall *p* < 0.001). Compared with asthma, participants with other lung diseases exhibited a significantly smaller R_5_ response (estimate −16.5%, 95% CI −24.8 to −8.2; *p* = 0.00010), and those with no lung disease showed an even lower response (estimate −20.9%, 95% CI −28.6 to −13.1; *p* < 0.000001). COPD and asthma–COPD overlap did not differ significantly from asthma.

Neither FeNO (estimate 0.002%; *p* = 0.98) nor baseline FEV_1_ (estimate −0.04%; *p* = 0.73) independently predicted ΔR_5_. Age was a modest negative predictor (−0.42% per year; *p* = 3.2 × 10^−6^), whereas BMI and sex were not associated with ΔR_5_.

### 4.5. Determinants of the ΔR_5_–ΔFEV_1_ Relationship

To assess whether the relationship between ΔFEV_1_ and ΔR_5_ differed across diagnostic groups, a model including a ΔFEV_1_ × diagnosis interaction was compared with a model assuming a common slope. No significant interaction was observed (F = 0.80; *p* = 0.52), indicating that the ΔFEV_1_–ΔR_5_ relationship did not differ meaningfully by diagnosis.

Although stratified analyses suggested variability in correlation strength across subgroups defined by diagnosis, FeNO, and baseline FEV_1_, formal interaction testing provided no evidence of slope modification by diagnosis or FeNO (*p* > 0.05). In contrast, baseline FEV_1_% predicted significantly modified the ΔFEV_1_–ΔR5 slope (β_interaction = −0.0317, *p* = 0.0028) (Table 4).

Correlations were negligible in participants with baseline FEV_1_ < 80% predicted, moderate in those with FEV_1_ 80–100% predicted, and strongest in those with FEV_1_ > 100% predicted.

### 4.6. Association Between Symptoms Following MCT and Physiological Responses

Self-reported pulmonary symptoms following methacholine challenge were progressively associated with larger physiological responses (Table 5). Participants reporting worse symptoms exhibited greater declines in FEV_1_ and larger increases in all IOS indices.

Across symptom categories, IOS parameters demonstrated larger relative stepwise changes between adjacent symptom groups than ΔFEV_1_, whereas ΔFEV_1_ showed a more gradual, approximately linear relationship with symptom severity.

Correlation analyses demonstrated moderate associations between symptom score and both ΔFEV_1_ (r = −0.35, *p* < 0.001) and ΔR_5_ (r = 0.32, *p* < 0.001). Formal comparison of dependent correlations confirmed that the linear association between symptom severity and ΔFEV_1_ was significantly stronger than that between symptoms and any IOS parameter (all *p* < 0.001), despite larger fold changes observed for IOS measures across symptom categories.

## 5. Discussion

In this large, clinically referred adult cohort undergoing methacholine challenge testing, we found only a weak-to-moderate inverse association between spirometric and oscillometric responses, supporting the concept that spirometry and oscillometry capture overlapping but distinct components of airway responsiveness. Although ΔFEV_1_ and ΔR_5_ were statistically associated, the explained variance was low, indicating substantial inter-individual variability. Although statistically significant, the modest correlation likely reflects the distinct physiological sensitivities of the two techniques rather than measurement inconsistency, with oscillometry capturing heterogeneous and peripheral airway responses that may evolve differently from expiratory flow limitation measured by FEV_1_. Importantly, a 20% decline in FEV_1_ corresponded on average to a 74% increase in R_5_, whereas the commonly used IOS threshold of ΔR_5_ ≥ 40% corresponded to only a modest decline in FEV_1_. Consistent with this physiological mismatch, IOS classified a substantially larger proportion of participants as responsive than conventional FEV_1_–PD_20_ criteria, and agreement between binary classifications was limited. Together, these findings argue against treating a 40% rise in R5 as physiologically interchangeable with FEV_1_–PD_20_ and instead support a complementary role for IOS alongside spirometry.

### 5.1. Relationship Between Changes in FEV_1_ and R_5_

The weak correlation between ΔFEV_1_ and ΔR_5_ is consistent with prior adult studies, where correlation coefficients are typically below 0.4 [8,9,11,12,15,17,18]. A notable exception has been reported in pediatric asthma cohorts, where stronger coupling between oscillometric and spirometric responses has been observed [16], potentially reflecting differences in lung size, airway mechanics, phenotype, and test performance.

A key extension of the present study is that baseline lung function modified the ΔFEV_1_–ΔR5 relationship. Stratified analyses showed negligible correlations in individuals with baseline FEV_1_ <80% predicted, with progressively stronger inverse correlations among those with preserved or supranormal baseline FEV_1_ [5]. Formal interaction testing confirmed effect modification by baseline FEV_1_% predicted (ΔFEV_1_ × baseline FEV_1_ interaction β = −0.0317; *p* = 0.0028), indicating that higher baseline FEV_1_ was associated with a steeper (more negative) ΔFEV_1_–ΔR_5_ slope—i.e., a larger ΔR_5_ increase for a given ΔFEV_1_ decline. This pattern suggests that when baseline expiratory flow limitation is minimal, oscillometric and spirometric responses may evolve more synchronously during bronchoconstriction. Conversely, in individuals with lower baseline FEV_1_, factors such as airway closure, gas trapping, heterogeneity of bronchoconstriction, and flow limitation may decouple impedance changes during tidal breathing from forced expiratory flow responses.

### 5.2. Increased Sensitivity of IOS Compared with FEV_1_

Our findings support prior observations that IOS, and R_5_ in particular, often identifies methacholine-induced airway changes at lower cumulative doses and in individuals who do not reach the conventional spirometric PD_20_ threshold [10,13,16]. In several adult cohorts, the provocative dose required to elicit a predefined oscillometric response has been reported to be lower than the dose required to induce a 20% decline in FEV_1_ [10,11]. In our cohort, the proportion classified as IOS-positive (ΔR_5_ ≥ 40%) was markedly higher than the proportion meeting FEV_1_–PD_20_ criteria, with limited agreement between methods. This discordance reinforces that IOS and spirometry should not be viewed as redundant measures of the same endpoint, but rather as complementary tests reflecting different physiological facets of methacholine responsiveness.

Although R_5_ was selected as the primary IOS metric for methodological simplicity and to reduce redundancy among correlated oscillometric variables, peripheral-airway–linked indices such as R_5_–R_20_ and reactance-derived measures (e.g., AX) may provide additional discriminatory information. Notably, in symptom-stratified analyses in the present study, these indices showed pronounced differences across symptom categories, consistent with a contribution from small-airway involvement during bronchial provocation. Reactance- and heterogeneity-sensitive indices such as X_5_ may further enhance detection of peripheral airway dysfunction, particularly in early or spatially heterogeneous bronchoconstrictive responses. A conceptual schematic summarizing of the physiological mechanisms underlying discordant oscillometric and spirometric responses during methacholine challenge testing is shown in Figure S1.

### 5.3. Thresholds: R_5_ ≥ 40% Versus FEV_1_ ≥ 20%

Despite frequent use of ΔR_5_ ≥ 40% as an IOS responsiveness threshold, consensus regarding its physiological equivalence to FEV_1_–PD20 remains lacking [4,19]. This threshold has been reported in prior bronchial challenge studies and provides a pragmatic reference for detecting methacholine-induced airway responsiveness; however, it should be interpreted as an operational criterion rather than a universally standardized physiological equivalent of PD_20_-FEV_1_. In our cohort, the mean ΔR_5_ associated with a 20% decline in FEV_1_ exceeded 40%, consistent with adult studies reporting larger R5 increases at spirometric PD_20_ [9,12,30]. Conversely, some cohorts—particularly pediatric studies—have reported smaller R_5_ changes for comparable FEV_1_ declines [13], and receiver operating characteristic analyses in selected datasets have proposed alternative R_5_ cutoffs [16,31]. Collectively, these findings suggest that a single universal ΔR_5_ threshold is unlikely to be interchangeable with PD_20_-FEV_1_ across ages, disease contexts, and measurement protocols. Rather than forcing equivalence, future work should focus on clinically anchored IOS response definitions and on identifying which oscillometric patterns best predict meaningful clinical outcomes. Importantly, thresholds derived from reference ranges in healthy populations may not correspond to clinically optimal cut-points. Outcome-based thresholds, determined by their ability to predict clinically meaningful endpoints, may differ depending on the intended application and represent an important direction for future research and clinical validati. From a clinical perspective, an IOS-positive response in the absence of a significant FEV_1_ decline should not be considered a false-positive result. Rather, it may reflect early or spatially heterogeneous airway narrowing, peripheral airway involvement, or bronchial hyperresponsiveness that does not produce sufficient expiratory flow limitation to reduce FEV_1_. When accompanied by symptom reproduction during testing, such findings may support physiologically relevant airway responsiveness and assist clinicians in interpreting equivocal bronchial challenge results.

### 5.4. Symptoms, IOS, and FEV_1_ During Methacholine Challenge

An important finding of this study is the relationship between patient-reported symptom worsening during methacholine challenge and physiological responses. Symptom severity was associated with both greater declines in FEV_1_ and larger IOS responses. However, ΔFEV_1_ showed a stronger and more linear association with symptom severity, whereas oscillometric indices demonstrated larger stepwise separations across symptom categories. Formal comparison of dependent correlations confirmed that the correlation between symptoms and ΔFEV_1_ was significantly stronger than that between symptoms and IOS parameters, despite larger fold changes observed for oscillometric measures across symptom strata. These results suggest that IOS may provide enhanced categorical discrimination across symptom states (stepwise separation), while spirometry tracks symptom severity more linearly at the cohort level.

These observations align with reports that some individuals develop respiratory symptoms and marked impedance changes during bronchial provocation despite limited spirometric change, including adult cohorts with irritant exposures and patients with asthma-like symptoms but negative spirometric criteria [17]. Together, these findings support the concept that oscillometry captures symptom-relevant airway mechanics not fully reflected by FEV_1_ alone. This divergence likely reflects fundamental physiological differences between the measurements: FEV_1_ reflects flow limitation and airway closure during forced expiration, whereas oscillometric indices obtained during tidal breathing capture frequency-dependent resistance and reactance that may be sensitive to heterogeneous and peripheral airway narrowing earlier in the bronchoconstrictive response.

### 5.5. Clinical Markers and IOS Responses

Associations between IOS responses and clinical markers were generally modest. In multivariable models adjusting for age, sex, BMI, baseline FEV_1_% predicted, and diagnostic category, FeNO was not an independent predictor of ΔR_5_ (Figure 3), suggesting that methacholine-induced oscillometric responses in this cohort were not explained by FeNO alone. Similarly, asthma control assessed by ACQ-6 showed limited association with IOS responsiveness, and IOS positivity did not differ meaningfully across ACQ-6 control strata (Table 1).

These findings indicate that methacholine-induced mechanical responses measured by IOS are largely independent of symptom control in daily life, reinforcing that airway hyperresponsiveness, symptom burden, and disease control represent related but distinct dimensions of airway disease. Taken together, our results show that oscillometry and spirometry capture complementary aspects of methacholine responsiveness: IOS identifies airway mechanical changes at lower levels of spirometric impairment and demonstrates greater stepwise separation across symptom categories, whereas ΔFEV_1_ shows a stronger linear association with symptom severity. These observations support the use of IOS as a valuable adjunct—rather than a replacement—to spirometry in the assessment of airway hyperresponsiveness in adults.

### 5.6. Strengths and Limitations

Strengths of this study include the large sample size and a uniform laboratory protocol with concurrent spirometry, impulse oscillometry, FeNO, and post-challenge symptom assessment, enabling a multidimensional characterization of methacholine responsiveness in a real-world referred adult cohort.

Several limitations merit consideration. First, the ΔR_5_ ≥ 40% criterion was applied as an empiric threshold and should not be interpreted as physiologically equivalent to the conventional FEV_1_–PD_20_ definition. Second, oscillometric responses may be influenced by device- and protocol-specific factors, which can limit generalizability across platforms. Third, the cross-sectional design precludes evaluation of test–retest reproducibility and prognostic value. ΔR_5_ was selected as the primary oscillometric endpoint to reduce redundancy among correlated IOS variables and to facilitate interpretability; however, reactance-based measures and small-airway–linked indices (e.g., AX, R_5_–R_20_) may provide additional information and warrant further study. Finally, diagnostic classification was based on clinical assessment and follow-up and may partly reflect information derived from the MCT itself, introducing potential circularity. The study was not designed to evaluate longitudinal clinical outcomes; therefore, outcome-based thresholds derived from receiver operating characteristic analysis could not be established. Future prospective studies linking oscillometric and spirometric responses to asthma control, exacerbation risk, and treatment response are needed to define clinically optimal cut-points.

### 5.7. Clinical Implications and Future Perspectives

From a translational perspective, these findings support incorporating oscillometry into methacholine challenge protocols to broaden physiological assessment of airway hyperresponsiveness—particularly in individuals with preserved baseline spirometry or modest FEV_1_ responses. In practice, ΔR_5_ may capture bronchoconstrictive changes during tidal breathing and identify responders who do not meet conventional FEV_1_–PD_20_ criteria.

Wider implementation will require (i) standardized IOS response definitions anchored to physiological reference frameworks, (ii) cross-device harmonization and calibration, and (iii) longitudinal validation of reproducibility and clinical utility. Ongoing technical standardization efforts in oscillometry provide an important foundation for these steps [5].

Future work should determine whether IOS responsiveness predicts clinically meaningful outcomes—such as exacerbations, symptom trajectories, or treatment response—beyond spirometry and established biomarkers, and should evaluate the value of IOS-guided strategies in pragmatic, multicenter study designs.

## 6. Conclusions

In this real-world adult cohort undergoing methacholine challenge testing, oscillometry and spirometry captured complementary, only partly overlapping aspects of airway responsiveness. IOS identified methacholine-induced mechanical changes at lower levels of spirometric impairment and showed pronounced stepwise differentiation across symptom severity categories, whereas ΔFEV_1_ exhibited a stronger linear association with symptom severity. These findings reinforce that airway hyperresponsiveness, symptom burden, and disease control represent related but distinct dimensions of airway disease and support the use of IOS as a valuable adjunct—rather than a replacement—to spirometry in bronchoprovocation testing. Broader clinical implementation will require standardized IOS response definitions, cross-device harmonization, and prospective studies to determine whether IOS-guided assessment improves diagnosis, risk stratification, and prediction of treatment response.

## Figures and Tables

**Table 1:** Baseline characteristics of 794 adults referred for methacholine challenge testing.

Characteristic ^1^	Asthma N = 360 ^1^	Asthma-COPD N = 35 ^1^	COPD N = 52 ^1^	OtherLD N = 155 ^1^	NoLD N = 192 ^1^	Overall N = 794 ^1^
Age (years)	49.1 ± 17.7	64.2 ± 15.1	64.8 ± 12.9	59.3 ± 15.5	50.3 ± 19.7	53.1 ± 18.3
Sex						
Female	228 (63%)	19 (54%)	28 (54%)	99 (64%)	123 (64%)	497 (63%)
Male	132 (37%)	16 (46%)	24 (46%)	56 (36%)	69 (36%)	297 (37%)
BMI (kg/m^2^)	26.4 ± 4.5	26.0 ± 3.7	25.5 ± 3.6	26.6 ± 4.7	26.0 ± 4.1	26.3 ± 4.4
Smoking status						
Never	237 (66%)	0 (0%)	6 (12%)	77 (50%)	106 (55%)	426 (54%)
Ex-smoker	107 (30%)	27 (77%)	33 (63%)	61 (39%)	74 (39%)	302 (38%)
Current	16 (4.4%)	8 (23%)	13 (25%)	17 (11%)	12 (6.3%)	66 (8.3%)
FEV_1_ (% predicted)	91.6 ± 12.3	82.5 ± 13.0	86.2 ± 13.8	91.9 ± 13.1	99.7 ± 12.8	92.9 ± 13.4
R_5_ (% predicted)	94.6 ± 23.0	89.8 ± 22.4	91.1 ± 24.5	88.5 ± 24.3	86.9 ± 22.0	91.1 ± 23.3
R_20_ (% predicted)	99.8 ± 21.8	89.4 ± 21.4	89.1 ± 21.1	92.3 ± 23.4	94.9 ± 23.4	96.0 ± 22.7
R_5_–R_20;_ (kPa/L/s)	0.03 (0.00–0.27)	0.04 (0.00–0.21)	0.04 (0.00–0.19)	0.03 (0.00–0.25)	0.02 (0.00–0.13)	0.03 (0.00–0.27)
X_5_ (kPa/L/s)	−0.09 (−0.41–0.00)	−0.10 (−0.25–−0.03)	−0.10 (−0.27–−0.03)	−0.09 (−0.28–−0.03)	−0.09 (−0.31–−0.01)	−0.09 (−0.41–0.00)
AX (kPa/L)	0.2 (0.0–3.6)	0.3 (0.0–1.9)	0.3 (0.1–2.5)	0.2 (0.0–2.9)	0.2 (0.0–1.4)	0.2 (0.0–3.6)
FeNO (ppb)	18.0 (0.0–312.0)	21.0 (0.0–107.0)	12.0 (0.0–48.0)	15.0 (0.0–68.0)	14.0 (1.0–82.0)	16.0 (0.0–312.0)
Asthma control (ACQ-6)						
Score < 0.75	120 (33%)	8 (23%)	19 (37%)	51 (34%)	88 (46%)	286 (36%)
Score 0.75–1.5	122 (34%)	15 (43%)	13 (25%)	51 (34%)	68 (36%)	269 (34%)
Score >1.50	117 (33%)	12 (34%)	20 (38%)	50 (33%)	35 (18%)	234 (30%)

^1^ Continuous data are presented as mean ± SD or median (min–max); categorical data as n (%). OtherLD: other lung diseases. NoLD: No lung disease. ACQ-6 reported as average score (sum of items divided by 6). ACQ-6 score <0.75: well-controlled asthma; score 0.75–1.50: partly controlled asthma; score >1.50: uncontrolled asthma; FeNO available in 792/794 and ACQ-6 in 789/794 patients.

**Table 2:** Methacholine responsiveness by diagnostic group. Mean spirometric and oscillometric responses and the proportion of participants meeting positivity criteria.

Diagnostic Group	n	Mean Fall in ΔFEV_1_, % ^1^	Fall in ΔFEV_1_ ≥20%^1^	Mean ΔR_5_, %	ΔR_5_ ≥ 40%	*p*-Value (FEV_1_ vs. R_5_)
All patients	794	15.7	37.4%	69.1	70.7%	<0.001
Asthma	360	19.0	51.1%	79.7	76.9%	<0.001
Asthma+COPD	35	20.0	60.0%	66.3	80.0%	0.096
COPD	52	17.0	46.2%	67.3	69.2%	0.038
Other lung disease	155	12.0	21.9%	59.1	60.6%	<0.001
No lung disease	192	11.2	17.7%	58.2	65.6%	<0.001

^1^ FEV_1_ positivity defined as ≥20% fall from baseline; IOS positivity defined as ≥40% increase in R5 from baseline. *p*-values from McNemar’s test comparing paired positivity rates (FEV_1_ vs. R5) within each diagnostic group.

**Table 3:** Cross-classification of methacholine challenge outcomes by IOS-PD_40_ and FEV_1_-PD_20_, (≤1440 µg).

FEV_1_-PD_20_	IOS Negative	IOS Positive	Total
Negative (>1440 µg)	165	331	496
Positive (≤1440 µg)	69	229	298
Total	234	561	794

Values indicate the number of patients classified as positive or negative by each criterion. *κ* = 0.09, *p* < 0.01.

**Table 4:** Correlation between changes in FEV_1_ and R5 during methacholine challenge, stratified by baseline FEV_1_ (% predicted).

Baseline FEV_1_ (% Predicted)	n	Pearson *r*	Spearman *ρ*
<80%	136	−0.043	−0.056
80–100%	420	−0.295	−0.284
>100%	238	−0.410	−0.449
Total	794		

Notes: ΔFEV**_1_** (%) was calculated as 100 × (post − baseline)/baseline, with negative values indicating a decline in FEV**_1_**; ΔR5 (%) was calculated as the percent change from baseline, with positive values indicating an increase in airway resistance. Correlations were computed within strata of baseline FEV**_1_** (% predicted).

**Table 5:** Pulmonary symptom severity after methacholine challenge and corresponding changes in spirometry and oscillometry (n = 791).

Symptom_Group	N	FEV1 Decline (%)	R5 Increase (%)	R5–20 Increase	AX Increase	Fres Rel. Increase (%)
(1) Much better	2	−5.1 (0.4)	48.0 (11.3)	0.10 (0.10–0.11)	1.11 (0.95–1.27)	6.1 (6.0)
(2) A little better	23	−10.4 (9.5)	47.6 (37.6)	0.08 (0.04–0.14)	0.95 (0.24–1.63)	4.2 (3.2)
(3) No change	212	−10.6 (8.6)	50.0 (37.2)	0.07 (0.03–0.16)	0.91 (0.20–2.30)	7.4 (8.5)
(4) A little worse	396	−16.8 (9.8)	72.2 (41.1)	0.14 (0.07–0.25)	1.85 (0.70–3.43)	11.5 (10.6)
(5) Much worse	158	−20.6 (9.4)	90.2 (48.3)	0.22 (0.12–0.34)	2.97 (1.48–4.84)	13.4 (10.6)

Notes: Values are mean (SD) for ΔFEV_1_, ΔR5, and ΔFres, and median (IQR) for Δ(R5–R20) and ΔAX. ΔFEV_1_ (%) was calculated as 100 × (post − baseline)/baseline (negative values indicate a decline). ΔR5 and ΔFres are percent changes from baseline (positive values indicate increases). R5–R20 and AX are reported as absolute changes from baseline.

## Data Availability

The data presented in this study are available from the corresponding author upon reasonable request, in accordance with institutional policies and GDPR requirements.

## Supplementary Materials

The following supporting information can be downloaded at: https://www.mdpi.com/article/10.3390/jcm15052025/s1, Figure S1: Conceptual mechanisms underlying discordant spirometric and oscillometric responses during methacholine challenge testing. (A) Peripheral airway narrowing increases respiratory system impedance and may be detected by oscillometry during tidal breathing before significant changes in FEV1 occur. (B) Heterogeneous bronchoconstriction produces ventilation heterogeneity and frequency-dependent resistance changes that further increase oscillometric responses. (C) During advanced bronchoconstriction, airway closure and expiratory flow limitation lead to a decline in FEV1, reflecting expiratory flow limitation measured by spirometry. (D) Differences in mechanical sensitivity and timing along the bronchoconstrictive response explain discordant IOS and spirometric outcomes, with oscillometric abnormalities emerging earlier and persisting as bronchoconstriction progresses.

## Abbreviations

ACQ-6	Asthma Control Questionnaire, 6-item version
AX	Reactance area (integral of negative reactance)
BMI	Body mass index
CI	Confidence interval
ERS	European Respiratory Society
FEV_1_	Forced expiratory volume in 1 s
FeNO	Fractional exhaled nitric oxide
Fres	Resonant frequency (of respiratory system impedance)
Hz	Hertz (frequency unit)
IQR	Interquartile range
IOS	Impulse oscillometry
IOS–PD40	IOS positivity defined as ≥40% increase in R_5_ (ΔR_5_ ≥ 40%)
κ (kappa)	Cohen’s kappa (agreement statistic)
MCT	Methacholine challenge test
mg/µg	Milligrams/micrograms (cumulative methacholine dose)
PC_20_	Provocative concentration causing a 20% fall in FEV_1_
PD_20_	Provocative dose causing a 20% fall in FEV_1_
PD_40_ (R_5_)	Provocative change criterion on IOS defined here as ΔR_5_ ≥ 40%
ppb	Parts per billion (FeNO units)
ρ (rho)	Spearman rank correlation coefficient
R_5_	Total respiratory system resistance at 5 Hz
R_20_	Proximal/central airway resistance at 20 Hz
R_5_–R_20_	Frequency dependence of resistance (often reflects small-airway contribution)
SD	Standard deviation
X_5_	Respiratory system reactance at 5 Hz
Δ (Delta)	Change (post − pre); e.g., ΔR_5_% is percent change in R_5_
