# Supplementary material for: Comparing Changes in FEV1 and Impulse Oscillometry Parameters Following Methacholine Challenge Testing: Physiological Correlates, Clinical Markers, and Pulmonary Symptoms
Source: J Clin Med. 2026 Mar 6;15(5):2025. doi: 10.3390/jcm15052025 (PMC12986344; doi:10.3390/jcm15052025)
Supplement: Supplementary file 1 [file jcm-15-02025-s001.zip › jcm-4173363-supplementary.pdf]

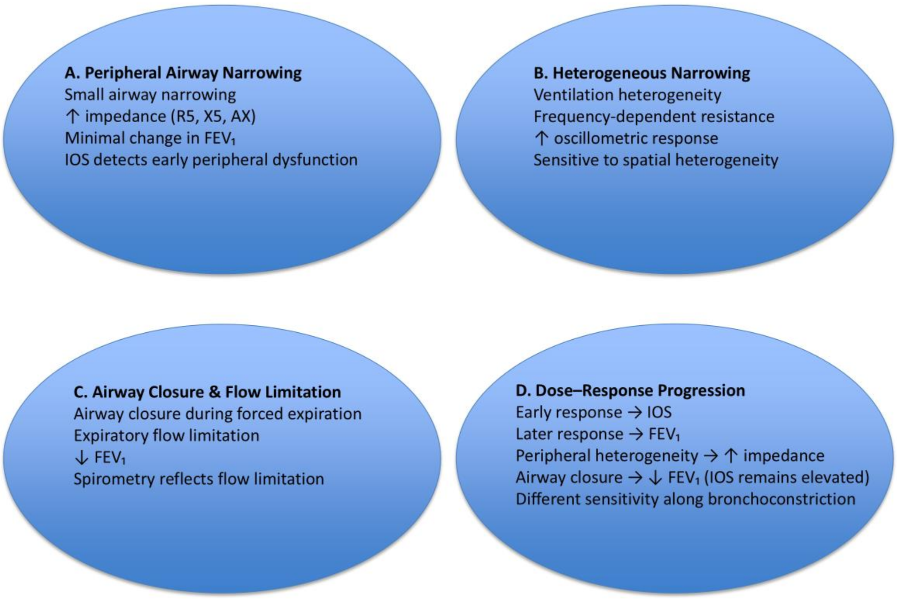

Figure S1. Conceptual mechanisms underlying discordant spirometric and oscillometric responses during methacholine challenge testing. (A) Peripheral airway narrowing increases respiratory system impedance and may be detected by oscillometry during tidal breathing before significant changes in FEV_1 occur. (B) Heterogeneous bronchoconstriction produces ventilation heterogeneity and frequency-dependent resistance changes that further increase oscillometric responses. (C) During advanced bronchoconstriction, airway closure and expiratory flow limitation lead to a decline in FEV_1, reflecting expiratory flow limitation measured by spirometry. (D) Differences in mechanical sensitivity and timing along the bronchoconstrictive response explain discordant IOS and spirometric outcomes, with oscillometric abnormalities emerging earlier and persisting as bronchoconstriction progresses.
